# Prevalence, contributory factors and severity of medication errors associated with direct-acting oral anticoagulants in adult patients: a systematic review and meta-analysis

**DOI:** 10.1007/s00228-021-03212-y

**Published:** 2021-12-22

**Authors:** Abdulrhman Al Rowily, Zahraa Jalal, Malcolm J. Price, Mohammed H. Abutaleb, Hind Almodiaemgh, Maha Al Ammari, Vibhu Paudyal

**Affiliations:** 1 School of Pharmacy, Institute of Clinical Sciences, College of Medical and Dental Sciences, Sir Robert Aitken Institute for Medical Research, University of Birmingham, Birmingham, B15 2TT UK; 2 Pharmaceutical Care Department, King Fahad Military Medical Complex (KFMMC), Medical Department, Ministry of Defence, Dhahran, Saudi Arabia; 3 Institute of Applied Health Research, University of Birmingham, Birmingham, UK; 4 NIHR Birmingham Biomedical Research Centre, University Hospitals Birmingham NHS Foundation Trust, Birmingham, UK; 5 Pharmaceutical Care Department, King Fahad Central Hospital, Jazan Health Affairs, Ministry of Health, Jazan, Saudi Arabia; 6 Pharmaceutical Care Department, King Abdulaziz Medical City, King Abdullah International Medical Research Center/King Saud Bin Abdulaziz University for Health Sciences, Riyadh, Saudi Arabia

**Keywords:** Non-vitamin K antagonist oral anticoagulants, Direct acting oral anticoagulants (DOACs), Medication errors, Systematic review, Meta-analysis

## Abstract

**Purpose:**

This study aimed to estimate the prevalence, contributory factors, and severity of medication errors associated with direct acting oral anticoagulants (DOACs).

**Methods:**

A systematic review and meta-analysis were undertaken by searching 11 databases including Medline, Embase, and CINHAL between January 2008 and September 2020. The pooled prevalence of errors and predictive intervals were estimated using random-effects models using Stata software. Data related to error causation were synthesised according to Reason’s accident causation model.

**Results:**

From the 5205 titles screened, 32 studies were included which were mostly based in hospitals and included DOAC treatment for thromboembolism and atrial fibrillation. The proportion of study population who experienced either prescription, administration, or dispensing error ranged from 5.3 to 37.3%. The pooled percentage of patients experiencing prescribing error was 20% (95% CI 15–25%; *I*^2^ = 96%; 95% PrI 4–43%). Prescribing error constituted the majority of all error types with a pooled estimate of 78% (95%CI 73–82%; *I*^2^ = 0) of all errors. The common reported causes were active failures including wrong drug, and dose for the indication. Mistakes such as non-consideration of renal function, and error-provoking conditions such as lack of knowledge were common contributing factors. Adverse events such as potentially fatal intracranial haemorrhage or patient deaths were linked to the errors but causality assessments were often missing.

**Conclusions:**

Despite their favourable safety profile, DOAC medication errors are common. There is a need to promote multidisciplinary working, guideline-adherence, training, and education of healthcare professionals, and the use of theory-based and technology-facilitated interventions to minimise errors and maximise the benefits of DOACs usage in all settings.

**Protocol:**

A protocol developed as per PRISMA-P guideline is registered under PROSPERO ID = CRD42019122996

**Supplementary Information:**

The online version contains supplementary material available at 10.1007/s00228-021-03212-y.

## Introduction

Direct-acting non-vitamin K antagonist oral anticoagulants (DOACs) including direct thrombin-inhibitor dabigatran, and two-factor Xa inhibitors rivaroxaban and apixaban have become the preferred choice in clinical practice for the primary and secondary stroke prevention in patients with atrial fibrillation, prevention and treatment of venous thromboembolism (VTE), and thromboprophylaxis in patients undergoing total hip or knee arthroplasty. Depending on indications, anticoagulation therapy can be given for a short term (up to around three months) or long term. Short-term anticoagulation therapy is most commonly indicated for primary perioperative prophylaxis of thromboembolic events such as those undergoing hip or knee replacement surgery. Long-term anticoagulant therapy with DOACs is recommended primarily for major cardiovascular conditions such as non-valvular atrial fibrillation (NVAF) [1].

DOACs have the advantage over Vitamin-K antagonists (VKA) of a much wider therapeutic window. While the relative ease of prescribing DOACs compared to VKAs makes them more commonly prescribed, therefore, healthcare professionals need to be aware of unwanted treatment outcomes associated with medication errors and suboptimal prescribing [1]. DOACs’ relatively shorter history of use precludes the current availability of long-term safety data. In addition, pharmacokinetic profiles and drug interactions are also not fully understood.

A risk and benefit profiling should be carefully considered/conducted before the prescribing of DOACs. The NICE guideline on atrial fibrillation recommends that bleeding risk, estimated using the HAS-BLED score, is taken into account when offering anticoagulation [1]. The HAS-BLED score estimates the risk of bleeding on a 9-point scale. Dose adjustments are often recommended with DOACs for renal creatinine clearance including contraindications for severe renal impairment [2].

Evidence of adverse events, particularly the incidence of bleeding related to the use of DOACs compared with vitamin-K antagonists in real-life patients, are beginning to emerge [3]. Previous systematic reviews of randomised controlled trials (RCTs) and observational studies have demonstrated the clinical benefits of DOACs compared to VKAs. DOACs significantly reduce the risks of intracranial haemorrhage, gastrointestinal, and major bleeding [4]. However, no direct head-to-head comparisons have been reported for each medicine of DOAC class. This leads to difficulty in the choice of drugs and dosage. A particular challenge is also the lack of availability of specific antidotes for all DOACs, and the lack of clear guidelines around treatment options for patients with intracranial bleeding and gastrointestinal bleeding under DOAC therapy [5].

Considering the above factors, DOACs are known to be one of the most common drug classes that are associated with adverse drug events (ADEs) [6]. The lack of long-term clinical experiences and the need for careful consideration of risk and benefit profiles makes DOAC candidates for medication errors, particularly prescribing errors which are often responsible for such ADEs [6].

To date, there exists no systematic reviews and meta-analysis that synthesise the prevalence of medication errors associated with DOACs including the prevalence of different types of medication errors. In addition, it is imperative to synthesise the contributory factors reported in the literature. Theoretical models are useful in identifying and interpreting factors that contribute to errors and to enable future interventions that can be effective in minimising such errors [7].

Reason’s accident causation model classifies errors into three different categories including (a) active failures which are unsafe acts committed by persons who are in direct contact with the patient or system and includes slips and lapses (errors in task execution), mistakes (errors in planning), and procedural violations (rule breaking); (b) error-provoking conditions within the workplace (e.g., time pressure, understaffing, inadequate equipment, fatigue, and inexperience); and (c) latent failures which arise from decisions made by policy makers, leaders, and top-level management [8]. This model has been widely adopted in research identifying the prevalence and causes of medication errors [9].

This systematic review aimed to determine the prevalence of medication errors associated with DOACs in clinical practice and to identify contributory factors associated with DOACs in adult patients using the Reason’s accident causation model. Results can enable healthcare professionals in diverse settings including primary, secondary, ambulatory care, and those in the interface such as community pharmacy to understand and mitigate common errors and associated consequences on patients and health systems.

## Methods

The systematic review protocol was developed in accordance with the Preferred Reporting Items for Systematic Review and Meta-Analysis Protocols (PRISMA-P) guidelines and registered with the International Prospective Register of Systematic Reviews (PROSPERO code is CRD42019122996). The review is reported according to the PRISMA guidelines [10] and MOOSE statements (Electronic supplementary material 1).

### Search strategy

A systemic search of the literature was undertaken using electronic databases: Medline, Embase, and Cumulative Index of Nursing and Cumulative Allied Health Literature (CINAHL), British Nursing Index (BNI), International Pharmaceutical Abstract (IPA), Cochrane Central Register of Controlled Trials (CENTRAL), and grey sources including Institute for Safe Medication Practices (ISMP), The FDA Safety Information and Adverse Event Reporting Program (FDA MedWatch), and Google Scholar databases from 2008 to September 2020. The search terms used included Medical Subject Headings and Natural Language Keywords for DOACs, namely dabigatran, rivaroxaban betrixaban, apixaban, and edoxaban and medication errors (specifically prescribing, and dispensing), and administration errors. Besides, each database search was restricted to English published studies (Electronic supplemental material 2) and conducted using the Boolean operators (AND, OR, and/or NOT). In addition, reference lists of included studies were screened to identify any additional relevant studies.

### Types of studies

We included studies which reported or investigated the rate of prescribing, administration, or dispensing errors associated with DOACs. Studies of adverse drug events that are not classified as errors were excluded, as were review articles, letters, opinion papers, and editorials.

### Types of participants

Adult patients (≥ 18 years) prescribed DOACs were eligible for inclusion.

### Outcomes

The primary outcome was the prevalence of medication errors associated with DOACs. Prevalence data on each error type i.e. prescribing, dispensing, and administration error was obtained by calculating the number of patients for whom an error was identified amongst the total number of patients included during the data collection period. The errors included in this study were those identified from the following source: chart review, medication review, and those reported to the error reporting systems. The secondary outcomes included the nature of causes, contributory factors, and severity of medication errors associated with DOACs.

### Study selection and data extraction

Two reviewers (AA and MHA) independently screened the titles and abstracts of all potentially relevant papers based on the selection criteria. This was followed by a full-text screening using Rayyan QCRI (a web and mobile app for a systematic review screening that facilitates collaboration between different reviewers for inclusion and exclusion of studies) [11]. Any disagreement about study inclusion was resolved through discussion with a third reviewer (VP). Duplicate independent extraction of data was undertaken by researchers working in pairs (AA, MHA, VP, ZJ). The data extracted included authors, year of publication, country and setting, study design, error prevalence, the nature of errors, error severity, and contributory factors. Data on study characteristics, error prevalence, and error causes were extracted. A meta-analysis was performed using Stata/IC 15.1 Software (StataCorp, College Station, TX, USA).

### Assessment of methodological quality

Quality assessment was undertaken by two independent reviewers (AA and MA) with disagreements resolved by consensus or referred to two other reviewers (HA, VP) as required using the critical appraisal skills programme (CASP) checklist [12].

### Data synthesis and statistical analysis

The meta-analyses were performed on the prevalence of medication errors associated with DOACs by a statistician (MP). A random-effects model was used to synthesise the data due to the expected heterogeneity between included studies, and the results obtained were presented using forest plots. Heterogeneity was described using *I*^2^ statistics and reporting of 95% prediction intervals [13]. The statistical significance of I^2^ was tested with chi-square test, and *P*-value level < 0.05 was set as the level of statistical significance. The effect size was calculated as the proportion with 95% confidence interval (CI).

Data on error causation were synthesized using Reason’s accident causation model [14] as a theoretical framework as per the classification of active failures, error-producing conditions, and latent failures.

## Results

### Search and study selection

**Fig. 1:**
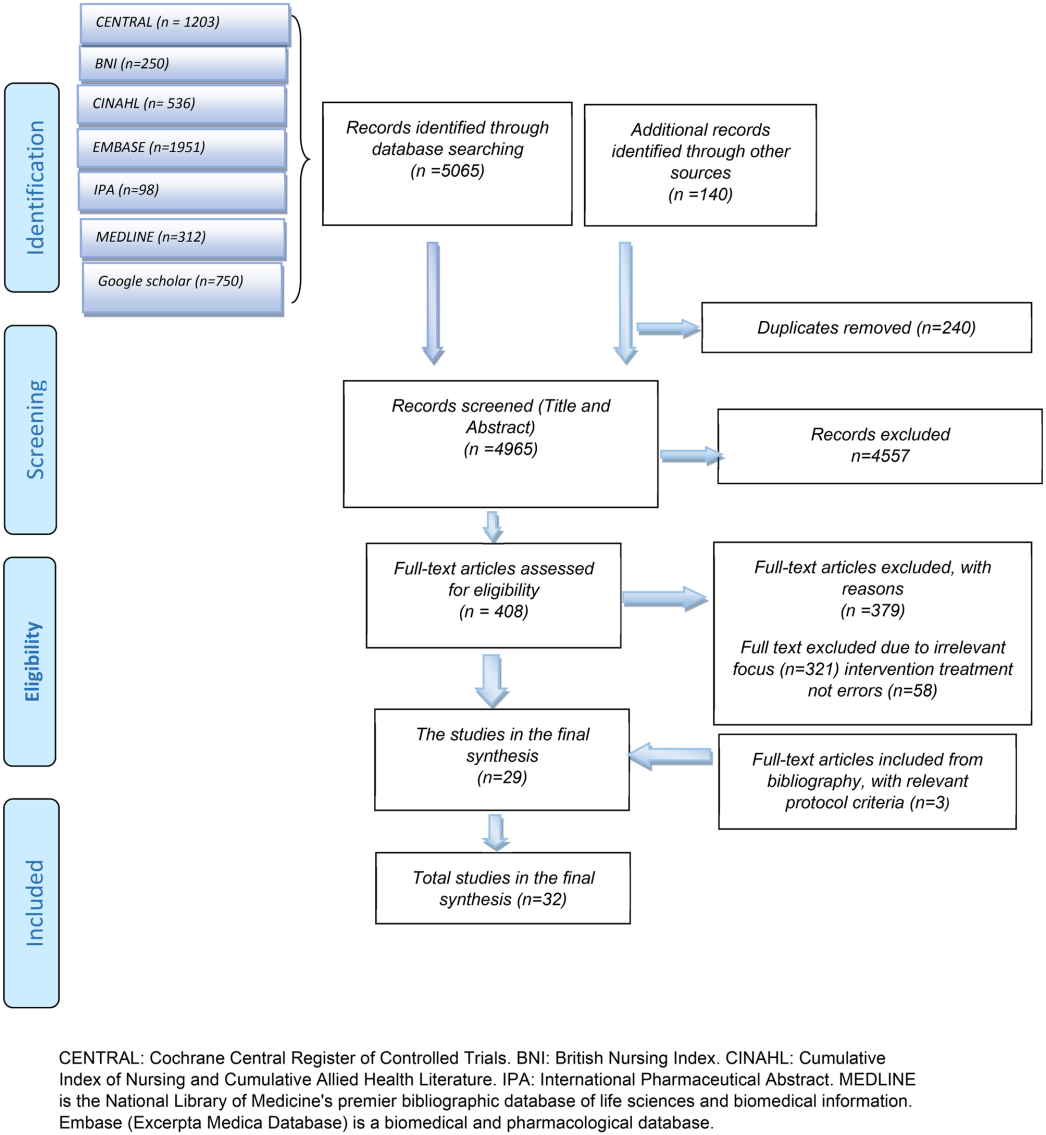
PRISMA flowchart describing systematic review search and study selection

The initial search resulted in 5205 titles search results across all the databases accessed (Fig. 1 shows the PRISMA flow diagram for this study). The duplicate results and studies that did not meet the inclusion criteria were identified and excluded. Overall, 408 articles were assessed for eligibility, of which 32 studies fulfilled the inclusion criteria for full-text review (Fig. 1) [15–46].

### Study characteristics

Out of the 32 studies, 12 (40.7%) were conducted in the USA [15, 19, 20, 24, 33, 35, 37–40, 42, 44]; three each in the UK (11.1%) [22, 34, 45] and France [18, 30, 46]; two each in Belgium [31, 36], Greece [27, 28], Australia [23, 41], Ireland [29, 32]; and one each in the Netherlands [21], Spain [43], Turkey [17], Israel [16], Denmark [25], and United Arab of Emirates [26]. Studies were conducted in university-affiliated or academic/teaching hospitals [15, 16, 18, 23, 25, 28–31, 34, 36, 37, 39, 41, 43, 45, 46], tertiary care non-teaching hospitals [24, 26, 33, 35, 38, 42], primary healthcare centres [22, 43], nursing home [19], private general hospital [27], pharmacist managed anticoagulation clinic [20], central medication registration [21], Poison control system [40], single center [32], and patient safety reporting system [44].

### Study quality

**Fig. 2:**
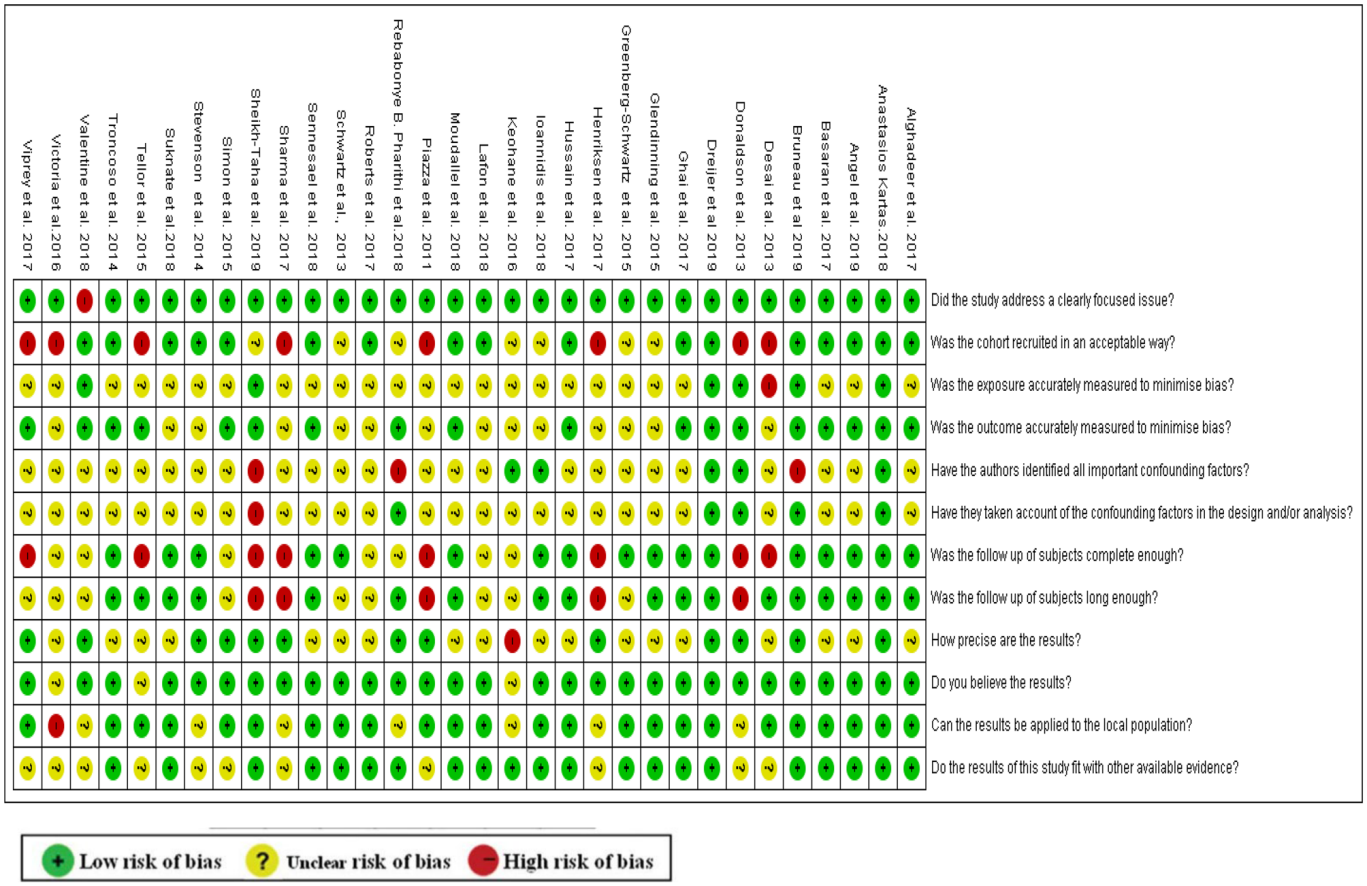
Quality assessment of studies included in the review

The quality of the included studies was variable (Fig. 2). Out of the 32 studies, only two studies (6%) met the eleven CASP-related quality assessment criteria for observational studies, while one study met 10 criteria. The key limitations centred on the lack of justification for the method of sampling and sample size, and exposures characteristics which include drugs and associated comorbidities were often poorly described (Fig. 2).

### Study design and data collection

About three quarter of the studies (*n* = 24) used retrospective routinely collected data [15, 16, 19, 21–26, 29, 31–35, 37–40, 42–46], six used prospective observational design [17, 18, 27, 30, 34, 36], while two studies used mixed study design which were combined with a retrospective chart review, prospective observational data, and clinical trial design [20, 28] (Table 1).

**Table 1:** Study characteristics with classification of medication error contributory factors

Author(s) Year	Country	Study design, Methods used to identify medication errors	Study setting	Study population	Sample size patient	Sample size (errors)	Types of medication error and data collection method	DOACs drug errors listed or investigated	indications		Results	Classification of contributory factors as per Reason’s Accident Causation model		
												Active Failure*	Error Provoking Condition**	Latent Condition***
Bruneau et al. [18]	France	Observational prospective study: multicenter	University hospitals	Elderly ≥65 years received and discharged on DOAC and Admitted to acute unit between February and July 2018	157		Prescribing error; retrospective review of medical records	Dabigatran, rivaroxaban, apixaban	AF, VTE, and Others		Prior to admission, (30.6%) had an inappropriate prescription. At discharge, (22.4%) had an inappropriate prescription; The nature of the inappropriateness was under- or overdosing (21.7%)	NR	NR	NR
Dreijer et al. [21]	The Netherlands	A cross-sectional study	Central Medication incidents Registration reporting system(CMR)	Medication error reported to Central Medication incidents Registration reporting system between December 2012 and May 2015	1000 medication error report		Prescribing error Administration error; errors reported to a national reporting system	Dabigatran, rivaroxaban, apixaban	AF		DOACs were the least frequently type of anticoagulant involved in the reports 3% Most anticoagulant medication errors were reported as prescribing errors (37.1%), followed by administering errors (29.8%). The majority of errors made in the prescribing phase arose from incomplete prescriptions. Omission errors were responsible for the highest percentage of errors in the administering phase	NR	NR	NR
Angel et al. [16]	Israel	Retrospective cohort study	University hospital	-	4427	1237	Inadequate treatment; retrospective review of medical records	Apixaban, rivaroxaban, dabigatran	AF		Among the 1237 patients with inadequate treatment, the most common types of errors were DOAC under-dosing (*n* = 578; 46.7%), VKA when DOAC was indicated (*n* = 258; 20.9%), DOAC despite contraindication to DOAC (*n* = 166; 13.4%), and DOAC over-dosing (*n* = 124; 10.0%).	Mistake: wrong dose	NR	NR
Valentine et al. [44]	USA	Retrospective reviewed database	Pennsylvania Patient Safety Reporting System database	Pennsylvania Patient Safety Reporting System database	1,811	1,546	Duplicate therapy; errors reported to a reporting system	Rivaroxaban, edoxaban, apixaban, dabigatran	AF, DVT, and PE		Of the 1,811 reports, 14.6% (n = 265) were considered ADEs and 85.4% (n = 1,546) were medication errors without harm.	NR	NR	NR
Sennesael et al. [36]	Belgium	Prospective observational study in the emergency departments	Teaching hospitals	Patients admitted with a thrombotic or bleeding event while under DOAC	46	38	Prescribing issues and inadequate monitoring; prospective medication review	Rivaroxaban, apixaban, dabigatran	NVAF, VTE		For the 46 patients taking DOAC, 38 adverse events were evaluated as serious ADRs. Among these, 20 ADRs (53%) were considered to be (potentially) preventable. Prescribing was the main stage of medication process involved in medication error (*n* = 16), followed by compliance (*n* = 5).	NR	Inadequate monitoring	NR
Moudallel et al. [31]	Belgium	Retrospective cohort study	University hospital	Hospitalized patients aged ≥60 years with at least one DOAC intake during hospital stay	772	193	Prescribing errors, inappropriate dosing; retrospective review of medical records	Dabigatran, rivaroxaban, apixaban	AF, VTE		Inappropriate dosing occurred in 25.0% of hospitalizations with 23.4, 21.9, and 29.7% for dabigatran, rivaroxaban, and apixaban, respectively (p = 0.084). Under dosing was most prevalent for apixaban (24.5%) compared to dabigatran (14.0%) and rivaroxaban (12.8%), p < 0.001. In 67.1% (apixaban), 26.7% (dabigatran), and 51.2% (rivaroxaban) of underdosed DOAC case	NR	NR	NR
Lafon et al. [30]	France	Prospective observational study:	University hospital	All subjects with DOAC admitted to the Limoges University Hospital ED	198	-	Prescribing errors (incorrect dosage according to age, renal function, co-medications); prospective observation of prescribing records	Rivaroxaban, dabigatran, apixaban	NVAF, VTE		In 16.2% of the cases, the treatment was not in according to the guidelines: 78% for prescribing errors (incorrect dosage according to age, renal function, co-medications) and 22 % for wrong initial indication	Mistake—wrong dose	Lack of experience •Insufficient education/training.	NR
Ioannidis et al. [27]	Athens, Greece	Prospective study: clinical pharmacists documented all cases where DOACs were prescribed	Private general hospital	Private general hospital	370	42	Prescribing errors (calculated DOACs dosage based on renal function); prospective observation of prescribing records	Rivaroxaban, dabigatran	AF, VTE, PE		A significant amount of patients (11.4%) received DOACs in a way that contradicts the product label guidelines.	•Mistake: wrong dose•Violation: doctor not writing the order not in time	NR	NR
Suknate et al. [41]	Australia	Retrospective review: The relevant data were collected by review of medical records by pharmacists	Teaching hospital	Teaching hospital	200	45	Prescribing error (the most common prescription error was under-dosing and overdosing; retrospective review of medical records	Rivaroxaban, apixaban, dabigatran	AF, VTE, PE		Inappropriate prescription of DOACs appears common, although not associated with complications. The prescription was deemed inappropriate in 45 patients (22.5%). The most common prescription error was under-dosing (for age, weight and renal function, or the indication), which was seen in 23 patients (11.5%). Overdosing was seen in seven (3.5%).	• NR	Lack of knowledge and experience	Lack of medication reconciliation service
Pharithi et al. [32]	Irland	Single centre, retrospective observational cohort study	Teaching hospital	AF patients who had received at least one dose of any of the NOACs	348	-	Prescribing error; retrospective review of medical records	Dabigatran, rivaroxaban, apixaban	AF			NR	NR	NR
Kartas [28]	Greece	Cross sectional study; Data extracted from MISOAC-AF registry	Tertiary care hospital	Adult patients with non-valvular AF or atrial flutter	768	-	Prescribing error; discharge medication review	Dabigatran, Rivaroxaban, Apixaban		AF	Off-label dosing (28.9%) was relevant to more than a quarter of NOAC users, while (23.8%) were underdosed and 21 (5.1%) were overdosed with NOAC	NR	NR	NR
Sheikh-Taha et al. [38]	USA	A retrospective study	Tertiary care center	Adult patients on NOAC between March 1 and June 30, 2017 Huntsville Hospital, Huntsville, Alabama, USA	909	-	Prescribing error; chart review	Dabigatran, rivaroxaban, apixaban, edoxaban		AF, DVT, PE	Almost 23.9% of the patients received doses inconsistent with the package labeling; 13.2% of patients received lower than recommended dosing, while 10.7% received higher than recommended dosing. The prevalence of inappropriate dosing was significantly more frequent among older patients, taking NOACs for AF(30.3%) compared to those using it for DVT/PE treatment (13%) The prevalence of inappropriate dosing was significantly higher in those with lower CrCl, and taking high number of medications	NR	NR	NR
Sharma et al. [37]	USA	Retrospective study: retrospectively reviewed electronic medical records of 41 patients. A clinical pharmacist collected this data.	Teaching hospital	Community-based hospital	41	10	Prescribing error; chart review	Rivaroxaban, apixaban	AF, VTE, and PE		Patients were dosed with 15 mg daily of rivaroxaban for DVT prophylaxis, which were corrected to 20 mg daily dose.	Mistake- wrong dose due to lack of renal dose adjustment and lack of initiation of anticoagulation	NR	NR
Viprey et al. [46]	France	Cross-sectional study: retrospective review; using data from medical records system of the Lyon teaching hospitals	University teaching hospitals	Hospitalized patients	1188	100	Prescribing errors—the appropriateness of the dosage of the drug; retrospective chart review	Dabigatran and Rivaroxaban	AF, DVT, and PE		The highest prevalence of DRPs was found among patients who received rivaroxaban for atrial fibrillation (14·6%; 95% CI, 10·7-18·5). A too low drug dose was the most frequent DRP (n = 56; 4·7%), followed by a too high drug dose (n = 37; 3·1%), contraindication (n = 5; 0·4%), and pharmacokinetic problem requiring dose adjustment (n = 2; 0·2%).	Mistake: wrong dose	NR	NR
Henriksen et al. [25]	Denmark	Descriptive study: retrospective review; three independent specialists in clinical pharmacology evaluated the severity of incident outcomes	University teaching hospitals	Reports to the Patient Safety Database; University Hospital	Not stated	147	Prescribing errors: excess or insufficient dosing; retrospective review	Dabigatran, Rivaroxaban	AF		Dabigatran: Total number within the subgroup (%);30 (21%); potentially Serious (%)—19 (63%)	Mistake: excess or insufficient dosing	System errors	NR
Alghadeer et al. [15]	USA	Retrospective review	University teaching hospitals	Patients that were prescribed dabigatran, rivaroxaban, or apixaban;	113	10	Prescribing error: lack of renal dose-adjustment in patients with reduced renal function was the most common reason for inappropriate use (for specific indication, renal function, age and/or weight); retrospective chart review	Dabigatran, rivaroxaban, and apixaban	STROKE, VTE		The dose of DOACs was unadjusted (for specific indication, renal function, age and/or weight) in 8.8% (*n* = 10) of patients collectively. All cases were due to unadjusted doses in patients with renal impairment and occurred in 9.2% (*n* = 6) of patients receiving dabigatran, 8.8% (*n* = 3) of patients receiving rivaroxaban, and 7.1% (*n* = 1) of patients receiving apixaban.	Mistake: duplicate therapy and wrong dose	Failure staff to follow policy and procedure. Inadequate laboratory results	NR
Hussain et al. [26]	UAE	Retrospective cross-sectional analysis	Tertiary care hospital	Patients who received dabigatran	61	28	Prescribing errors; retrospective review of medical records	dabigatran	NVAF		Inappropriate dose was administered in 7 of the 61 patients prescribed dabigatran.	NR	Inadequate knowledge off label indication	NR
Ghai et al. [22]	UK	Retrospective review: data was collected from two GP practices in BognorRegis.	Primary care hospital	Patients with NVAF who prescribed DOACs	73	-	Documentation error and prescribing and monitoring errors; retrospective review of medical records	Rivaroxaban	NVAF		12 patients despite having impaired renal function (CrCl < 50) were prescribed the higher dose. Patients with impaired renal function (CrCl < 60) did not have their renal function monitored more frequently as is suggested by NICE	Mistake: wrong dose	•Lack knowledge of how to adjust dose CrCls •Poor communication between team members	Lack of training
Basaran et al. [17]	Turkey	Prospective, observational study: patients with NVAF were screened for OAC prescription	University teaching hospitals	Patients with NVAF; outpatient cardiology clinics	148	24	Inappropriate prescribing; prospective review of prescribing records	Dabigatran, rivaroxaban	NVAF		Inappropriate drug use is frequent among patients with DOACs.	Mistake: wrong dose	NR	NR
Roberts et al. [34]	UK	Prospective observational study:	University teaching hospitals	Patients attending AF clinics, acute medical and cardiology wards; teaching hospital	190	41	Prescribing errors (incorrect dosage according to age, renal Function); prospective review of prescribing records	Rivaroxban, apixaban, edoxaban	AF		Apixaban had the highest rate of inappropriate dosage. As most prescribing errors involved inappropriate dose reduction	•Slips and lapse-memory •Mistake- wrong dose	NR	NR
Victoria et al. [45]	UK	Retrospective review: The data was collected for all DATIX system-reported incidents by clinical pharmacists	University teaching hospitals	-	-	25	Prescribing errors(missed dose, wrong dose for indication, incorrect dosage according to age, renal Function); incident reporting system	Rivaroxaban , apixaban, dabigatran, edoxaban	AF		Patients were prescribed the wrong dose for indication, e.g., AF dose of Apixaban for PE. DOACS were often not available on the ward and patients went as long as 48 hours without anticoagulation	Slips—memory lapses •Lapse: wrong correct label	Lack of knowledge and familiarity with DOACs	NR
Keohane et al. [29]	Ireland	Cross-sectional data was collected from inpatients over a 3-week period	University teaching hospitals	Internal medicine and cardiology wards)	30	-	Prescribing errors (inappropriate dose, indication); review of medical records	Rivaroxaban, apixaban and dabigatran	AF		Out of 70% of the patients, almost 10% were on a NOAC for an inappropriate indication and 11% on an inappropriate dose for the CrCl - Potential drug interactions were common, with 63% of patients concomitantly taking a cautioned or contraindicated medication.	Mistake-prescribing for wrong indication and lack of dose adjustment	NR	NR
Glendinning et al. [23]	Australia	Retrospective review: hospital pharmacy provided a list of patients dispensed either apixaban or rivaroxaban; The medication charts and progress notes of these patients were reviewed for prescribing errors and the presence of any subsequent complications	University teaching hospitals	-	250	-	Prescribing error, documentation error; medical charts and progress notes	Rivaroxaban, apixaban	VTE		19.5% of medication charts prescribing DOACs contained errors	NR	NR	NR
Tellor et al. [42]	USA	Retrospective review	Tertiary community hospital	Patients received at least one treatment dose of rivaroxaban	714	445	Prescribing errors: inappropriate dose; retrospective chart review	Rivaroxaban	NVAF, PE, DVT		Of the 445 patients evaluated, 36.9% of patients treated for NVAF and 12.4% treated for VTE were on an inappropriate regimen. The most common errors in the rivaroxaban regimen for VTE treatment were an inappropriate dose (8 patients, 5.7%)	NR	NR	NR
Simon et al. [39]	USA	Retrospective review: a search of the electronic health record (EHR) was conducted	Academic medical centre	Patients seen in outpatient clinics	395	249	Inappropriate prescribing, patient-reported inappropriate use; retrospective chart review	Apixaban, dabigatran, rivaroxaban	NVAF, PE, DVT		Of contacted patients taking rivaroxaban, 24 (23%) reported taking it inappropriately without food, and of contacted patients taking dabigatran, six patients (14%) endorsed inappropriate storage of dabigatran. Ten patients (6%) reported missing at least one TSOAC dose per week.	Mistake- wrong dose and failure to give rivaroxaban without food	NR	NR
Greenberg-Schwartz et al. [24]	USA	Retrospective review	Community hospital	-	-	-	Errors included inaccurate renal and hepatic dosing adjustments, incorrect dosage based on indication and duplication of anticoagulation agents; retrospective review of medical records	Rivaroxaban	NVAF, PE, DVT		Errors included inaccurate renal and hepatic dosing adjustments, incorrect dosage based on Indication and duplication of anticoagulation agents. Educational program “LEARN” reduced the error from 31.7 to 22%	Slips- acronym errors •Lapse—duplicate therapy	NR	Insufficient education/training opportunities
Stevenson et al. [40]	USA	A retrospective review and prospective observational case series; Data for cases were collected by different poison system staff members	Poison control center	Dabigatran, rivaroxaban exposures into the California Poison Control System	Not stated	49	Therapeutic error: patient mistakenly ingested or was given another individual’s medication; retrospective and prospective review of medical records	Dabigatran and rivaroxaban	DVT		There were 7 cases of dabigatran accidental extra dosing. The excess doses ranged from 75 to 750 mg	NR	NR	NR
Troncoso et al. [43]	Spain	Observational study: Retrospective review; electronic clinical records	Primary healthcare centres	Patients with AF who have been prescribed dabigatran and rivaroxaban	2324	197	inappropriate prescribing; retrospective chart review	Dabigatran, rivaroxaban	AF		Some patients had not been prescribed dabigatran or rivaroxaban even though they were potentially suitable candidates for these drugs.	Slip-wrong dose and wrong choice dose	NR	NR
Donaldson et al. [20]	USA	began as a retrospective review of patients on dabigatran therapy and continues as a prospective, intention-to-treat analysis, completed by a pharmacist-managed anticoagulation clinic	Anticoagulant clinics	Patients on dabigatran therapy; Pharmacist managed anticoagulation clinic	221	54	Prescribing errors; retrospective chart review	Dabigatran	VTE, PE, stroke		Of the 54 patients experiencing an ADE, five patients (9.3%) should have been on a lower dose based on renal function and/or concurrent drug interactions.	NR	NR	NR
Schwartz et al. [35]	USA	Retrospective review	Community hospital	-	-	-	Prescribing errors (incorrect dosage according to age, renal function) Inappropriate indication-inappropriate time of administration (dietary interactions); retrospective review of medical records	Dabigatran	AF		Educational activities “CARE” reduce prescribing error from 40% to 28%.	NR	NR	NR
Desai et al. [19]	USA	Cross-sectional: retrospective review; The medication error reports in MEQI are collected by healthcare professionals	Nursing Home	Individual medication error incidents reported by North Carolina nursing homes to the MEQI	Not stated	1623	Prescribing, documenting or Monitoring errors; retrospective chart review	Dabigatran	AF		Anticoagulant errors were more likely to be associated with patient harm (2% vs 1%, *p* = 0.001) compared to all other errors.	Slip and lapse-pharmacy dispensing issue and drug name confusion, incorrect transcription.	•Inadequate knowledge •Lack communication •Distraction, •Work overload •failure staff to follow policy and procedure and inadequate information •Shift change	NR
Piazza et al. [33]	USA	Retrospective review: physicians, pharmacists, and a hospital patient safety officer reviewed all reported anticoagulant-related events	Tertiary care Hospital	Inpatient anticoagulant-associated medication errors;	Not stated	226	Transcription errors: missed medication doses; retrospective chart review	Not stated	AF, DVT		Of 463 anticoagulant-associated ADEs, 226 were medication errors (48.8%), 141 were ADRs (30.5%), and 96 (20.7%) involved both a medication error and ADR	Slips and lapse: transcription errors Mistakes: wrong medication prescribed for the indication	NR	NR

*ADEs* adverse effect events, *ADR* adverse drug reaction, *AF* atrial fibrillation, *CrCl* creatinine clearance, *DOACs* non-vitamin K antagonist oral anticoagulants, *DRP* drug-related problem, *DVT* deep vein thrombosis, *MEQI* Medication Error Quality Initiative, *NR* not reported, *NVAF* non-valvular atrial fibrillation, *OAC* oral anticoagulants, *TSOAC* target specific oral anticoagulants, *VKA* vitamin K antagonist; *VTE* venous thromboembolism

^*^Active failures are unsafe acts committed by people who are in direct contact with the patient or system. They take a variety of forms including slips and lapses (errors in task execution), mistakes (errors in planning), and procedural violations (rule breaking)

^**^Error-producing conditions within the workplace (e.g., time, pressure, under staffing, inadequate equipment, fatigue and inexperience)

^***^Latent failures which arise from decisions made by policy makers, leaders, and top-level management

### Adopted definitions of medication errors

Although a clear definition of medication error was not provided in most of the included sample studies (*n* = 30), two studies used established definitions used by North Carolina’s Medication Error Quality Initiative (MEQI), and National Coordinating Council for Medication Error Reporting and Prevention (NCC MERP) [19, 37].

### Prevalence of errors

Proportion of patients who experienced either prescribing, dispensing, or administration errors across the studies ranged from 5.3 to 37.3%. The variability in the rates of errors was often contributed to the range of DOACs being indicated, types of DOACs agent used in the study settings, and consideration of either certian type of error or range of errors across the different stages of medication use process (Table 1).

### Prescribing errors and contributory factors

**Fig. 3:**
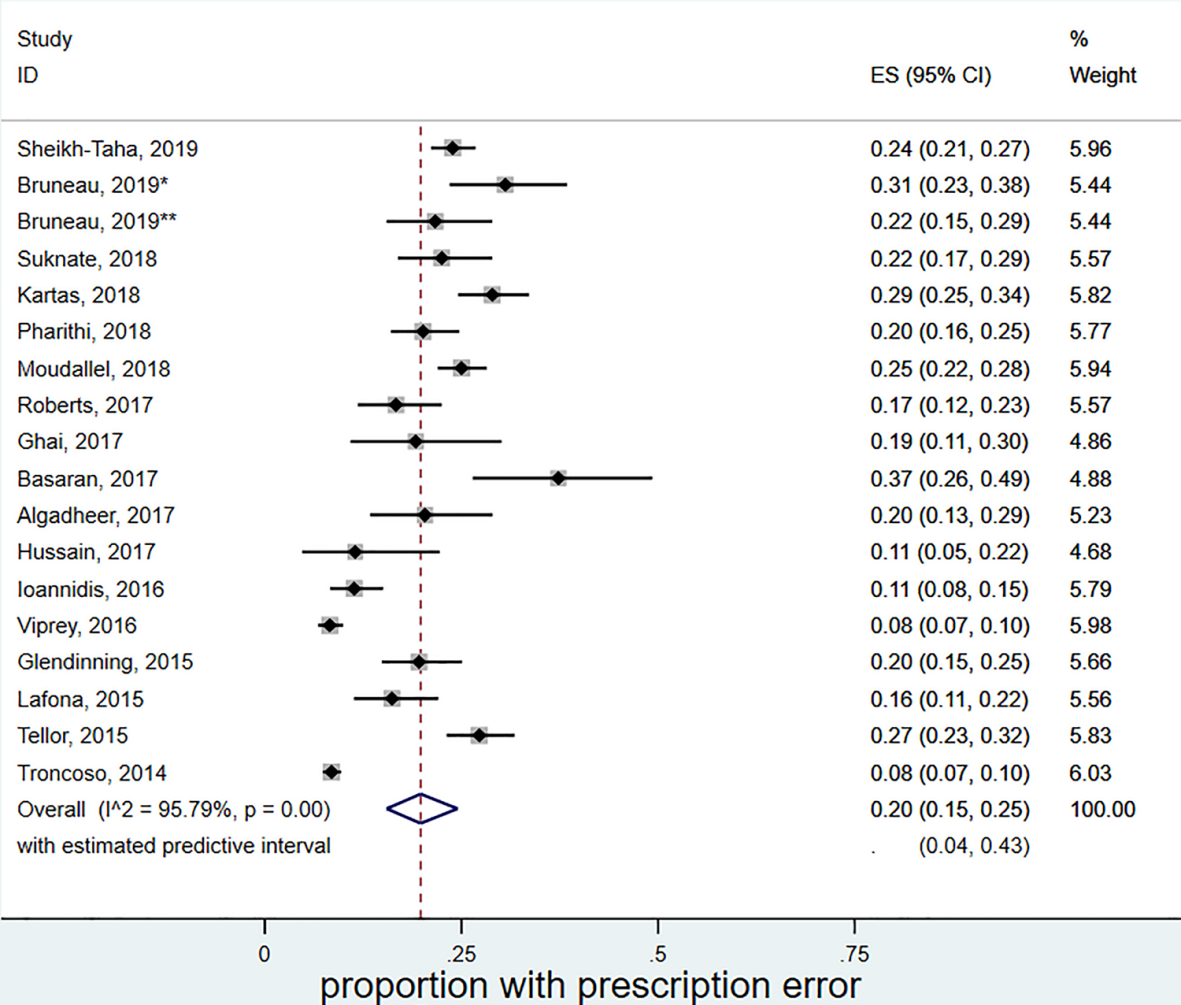
Forest plot meta-analysis of the prevalence of prescribing errors amongst all patients prescribed DOACs. *Pre-admissions; **at discharge. ES (95% CI): proportion with 95% confidence interval (CI), p-value is from a Chi-square test for heterogeneity

Eighteen studies reported the number (proportion) of patients amongst the study population where prescribing errors were identified. The overall proportion of patients experiencing prescribing error was found to be 20% (95%CI 15–25%) with a 95% prediction interval (95% PrI) between 4 and 43% (Fig. 3).

**Fig. 4:**
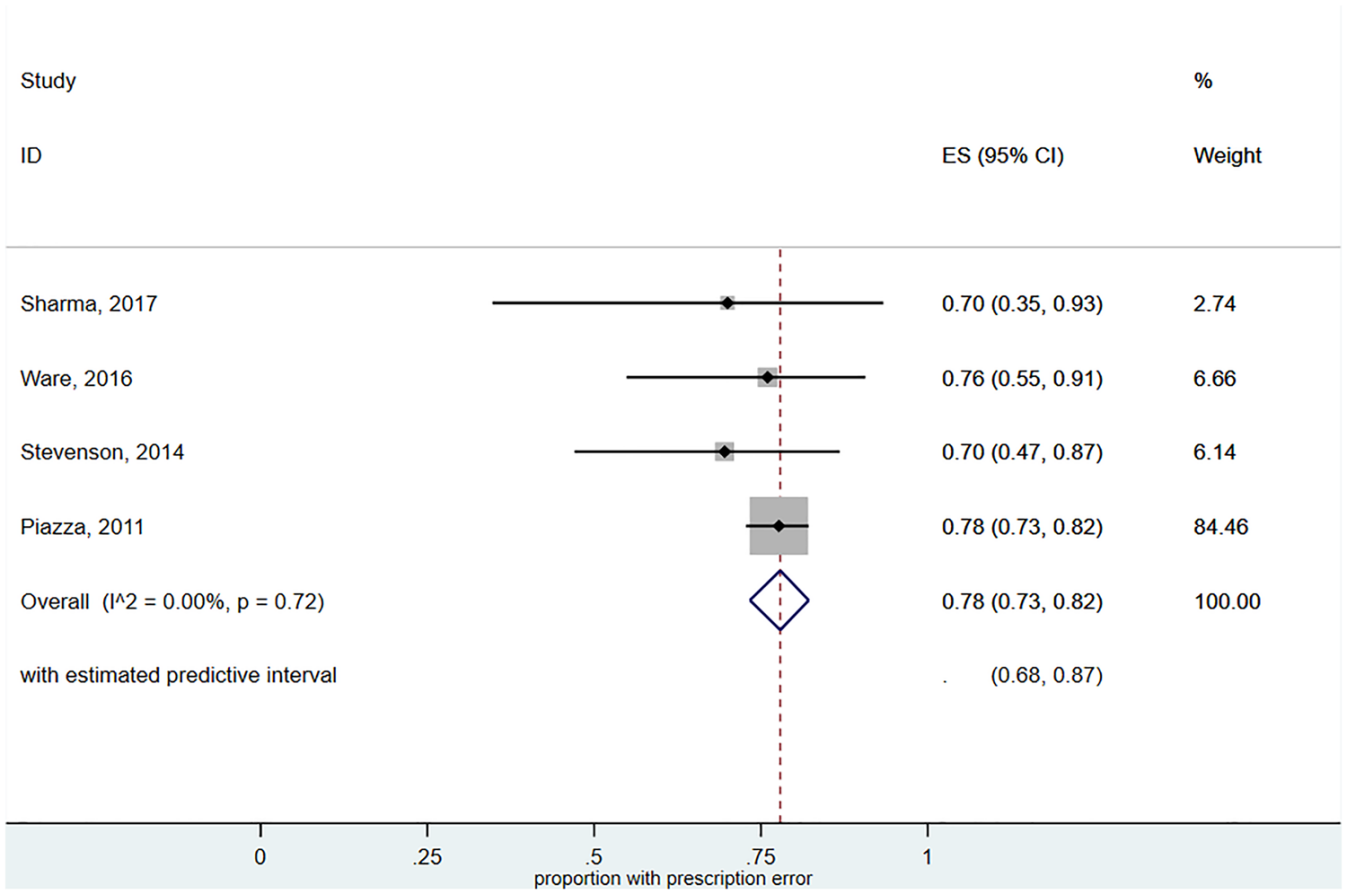
Forest plot meta-analysis of the prevalence of prescribing errors amongst all medication errors associated with DOACs. ES (95% CI): proportion with 95% confidence interval (CI), p-value is from a Chi-square test for heterogeneity.

Prescribing errors was the most commonly reported error types. The proportion of prescribing errors as a proportion of all error types ranged from 70 to 78%. The pooled estimate of error rate was 78% (95%CI 73–82%; *I*^2^ = 0, 95%PrI 68–87%) (Fig. 4).

Prescribing errors detected in the studies included related to over or under dosing [15], missed medication doses [18], and inappropriate prescribing in the context of indication, altered renal function (particularly creatinine clearance), advanced age, altered weight, concomitant medications, and prescribed contraindication based on product label guidelines (Table 1). Studies also reported duplicate therapy from different DOAC agents as one of the common prescribing errors.

A study conducted in a tertiary care community hospital in the USA reported that over a third of patients (*n* = 92, 35.4%) who received rivaroxaban had an inappropriate dose ordered. Forty one of these patients were considered too low, while 51 were considered too high based on the patient’s renal function at the initiation of rivaroxaban [42]. A UK study showed that 12 (16.4%) of the included patients were prescribed the full dose despite having impaired renal function (CrCl < 50) [33]. In a study conducted in Ireland, an inappropriate dose according to CrCl was found in 11% [29]. The study conducted in a community hospital by Tellor et al. to evaluate the appropriateness of rivaroxaban’s dosing, indication, and safety reported that 36.9% of patients treated for NVAF and 12.4% treated for VTE were on an inappropriate regimen [42]. Furthermore, twenty patients (7.7%) were treated for NVAF with an unapproved 10 mg dose of rivaroxaban, whereas two patients (0.8%) had an inappropriately high dosing frequency of twice daily. Although rivaroxaban is only approved for NVAF, six patients (2.3%) were treated for atrial fibrillation as off label use [42].

### Administration error

**Fig. 5:**
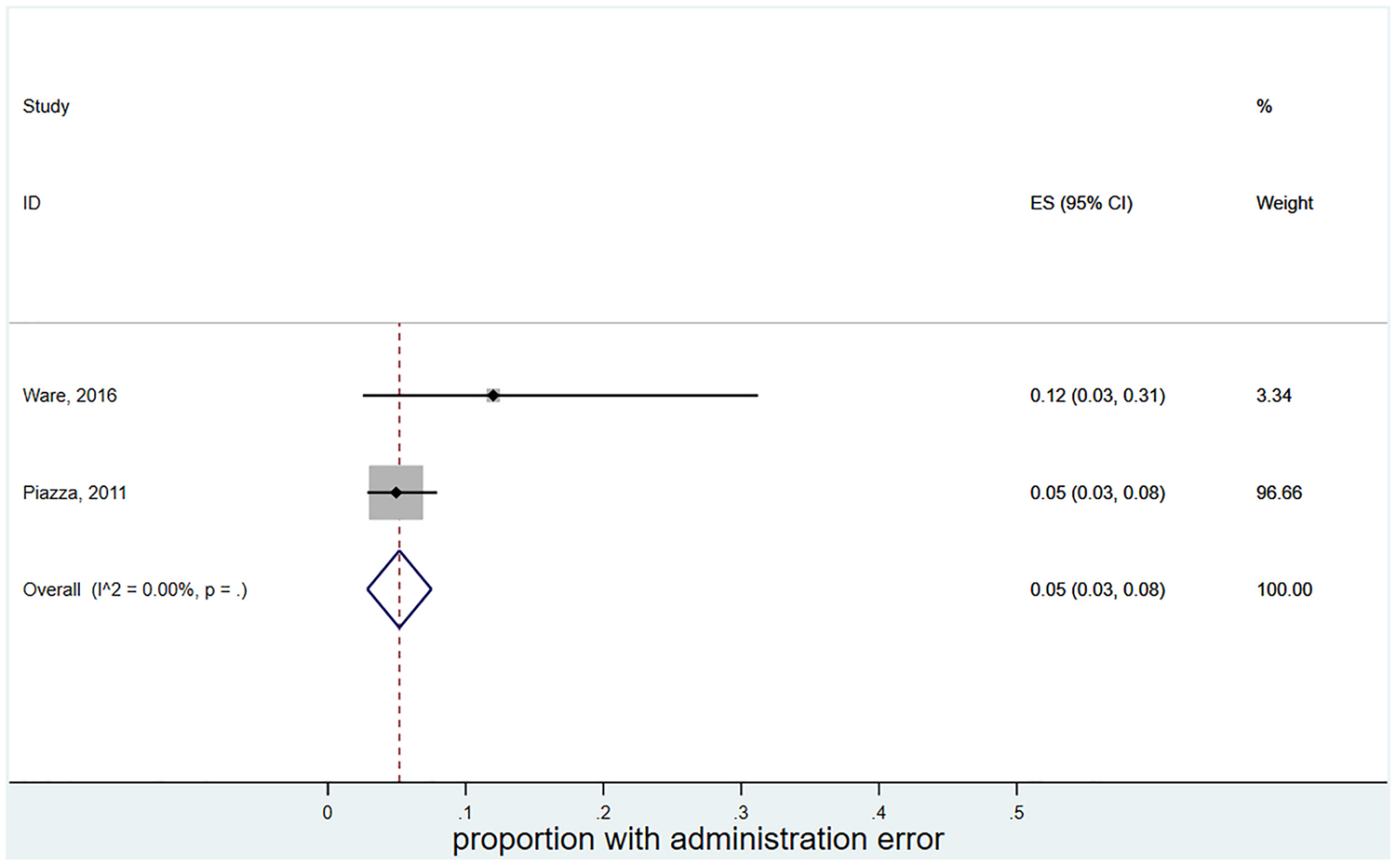
Forest plot meta-analysis of the prevalence of administration errors amongst all medication errors associated with DOACs. ES (95% CI): proportion with 95% confidence interval (CI), p-value is from a Chi-square test for heterogeneity

Only two studies reported the rate of administration errors amongst all types of medication errors associated with DOACs. Heterogeneity was low (*I*^2^ = 0), though there were little data to estimate between-study variance. The pooled estimate gave a prevalence of errors to be 5% (95% CI 3–8%). However, nearly all of the weight in the meta-analysis is given to one study [33]. Therefore, there were insufficient studies to calculate a prediction interval (Fig. 5).

A prospective study conducted at a private general hospital in Greece reported that Apixaban was the most frequent DOACs agent to be erroneously administered (13 of 76 cases, 17.1%), followed by rivaroxaban (28 of 257 cases, 10.9%) and dabigatran (one of 37 cases. 2.7%) [27]. One study reported that DOACs were often unavailable on the ward and patients went as long as 48 h without anticoagulation while admitted [45]. Another study reported that 24 patients (23%) were taking rivaroxaban inappropriately without food, and six patients (14%) endorsed inappropriate storage of dabigatran [39]. A study by Stevenson et al. described an administration error related to dabigatran, in which patients mistakenly ingested the drug or were given another patient’s medication [40].

### Dispensing errors

The prevalence of dispensing errors amongst all medication errors associated with DOACs was described in one study. This study reports that out of 25 total prescribing errors associated with DOACs, only 2 (8%, 95% CI = 0–18.6%) were identified as dispensing errors [45].

### Subgroup analyses

Subgroup analyses of error prevalence across various indications and study settings are provided in electronic supplementary material 3.

### Consequences, severity of errors

Only four included studies [15, 25, 40, 44] assessed the severity of harm associated with the error. Haemorrhage was the most frequently reported adverse event associated with the errors especially in patients with lower CrCl and older age. For instance in a US study, haemorrhage occurred in 70.2% of the included subjects and of whom almost 40% of the patients were 80 years or older; furthermore, mortality reported in two patients in this study [44]. However detailed causality assessment linking errors with adverse events were missing. In another study in Danish patients prescribed dabigatran, two incidents of potentially fatal outcomes were also reported [25].

### Contributory factors

Of the 32 studies, only 27 reported contributory factors associated with medication errors related to DOACs, yet none of these 27 studies used any theory (e.g., behavioural or organisational) in their methodology or data collection or analysis. The results from these studies were categorised according to Reason’s accident causation model and showed in Table 1. The most commonly reported contributing factors were active failures and mistakes, which included failure to consider risk factors of creatinine clearance, age, or weight when prescribing such medicines. In addition to the inexperience and lack of knowledge of the prescriber, lack of inter-professional collaboration and poor communications with other healthcare providers or with the patients, as well as staffing and workload issues, were also reported as contributory factors that lead to medication errors associated with DOAC (Table 1).

## Discussion

Oral anticoagulants are currently among the most widely prescribed medications in clinical practice, with DOACs becoming more and more utilized over VKAs. DOACs have more predictable pharmacokinetic profiles, lower bleeding risks, and fewer drug interactions than warfarin [47]. Complexity of patients’ conditions and polypharmacy therapeutics on the other hand can increase the risk of adverse events. The popularity of DOACs and their associated adverse events made them of interest to investigate errors and identify their contributing factors. The aim of this study was to systematically review the prevalence of medication errors associated with DOACs and its contributory factors. Studies reported a wide prevalence rates often influenced by the method used for error definition and detection, drugs being investigated for errors, and the patient population.

### Prevalence of errors

This systematic review found that prescribing errors, particularly the dose-related, were the most prevalent type of errors associated with DOACs. Although DOACs are given in fixed doses and not requiring routine coagulation monitoring, dosing varies based on drug used, indication, renal function, age, and body weight, as well as concurrent medications [48–52]. The nature of errors identified in the systematic review was reflective of these factors.

### Severity of errors

The severity of medication incident reports may help identify possible areas for improvement in reporting the adverse events and types of errors related to DOACs. Few studies had reported the severity of adverse events associated with medication errors. New thrombotic and bleeding events risks are found associated with suboptimal prescribing [53], while the likelihood of hospitalisation and mortality increases with overdosing. Detailed causality assessments were however often missing in the included studies as to whether the errors primarily contributed to the adverse events.

### Implications for practice

There are several risk reduction strategies relevant to minimise and avoid harm related to medications errors with DOACs. Development of novel-theory based and technology-enabled interventions can improve patient safety. Education of healthcare professionals through training sessions and adopting anticoagulant stewardship programme can be effective. Secondly, undertaking medication reconciliation on admission and discharge as well as upon care transfer in combination with medication reviews. 

The relative ease of prescribing and monitoring DOACs compared to VKAs makes them first prescribing choice for many indications like a non-valvular atrial fibrillation and VTE. Such prescribing preference could lead to increased incidence of different types of prescribing errors. Each DOAC has a different dosing schedule and dose adaptations, mostly reductions, depending on one or more patient-specific factors including age, weight, renal function, indication, and concomitant medications. As seen in the included studies, one of the reasons for overdosing DOACs was failing to adjust the dose of DOACs for specific indication, renal function, age, and/or weight. Similarly, elderly patients who are at high risk of developing stroke are often likely to be underdosed. Amongst the included studies which reported nearly fatal or fatal adverse events linked to prescribing errors, severe incidents most commonly occurred during sector change such as admissions, discharge, or undergoing surgery [25, 44]. It is imperative that additional precautions be applied during patient transitions across sectors as well as prior to and after surgical procedures.

This review has shown that the contributing factors to medication errors with DOACs are multifactorial. These factors include inappropriate drug selection and lack of dose adjustments consideration due to failure to approach patients holistically by assessing, for instance, renal function, medical, or medication histories or demographics. Inexperience, poor communication, and lack of inter-professional collaborative practice together with non-compliance to clinical guidelines also contributed to their inappropriate prescribing. Therefore, adopting inter-professional team-based clinical practice and leaning as well as pharmacist-led anticoagulant stewardship program are likely to minimise errors. A recent meta-analysis showed that including a pharmacist in clinical rounds alongside educational interventions and prescription reviews can significantly reduce prescribing errors by as much as three quarters [54].

### Implications for research

Future studies should expand on the current research to determine techniques to reduce the occurrence of the more prevalent errors associated with DOACs. Behavioural frameworks such as the theoretical domains framework (TDF) are useful in identifying target behaviours and future interventions [55]. Future studies should consider data from non-hospital settings and undertake rigorous causality assessment to investigate the link between errors and adverse outcomes.

### Study strengths and limitations

To the best of our knowledge, this is the first systematic review and meta-analysis that aim to review the prevalence of DOACs associated errors and its contributed factors. A wide range of databases was used including grey literature. However, the current review was limited to the literature published in English language. Overall, the review endorsed the variability of clinical implications and consequences of errors based on patients’ characteristics such as age, co-morbidities, and concomitant of drug therapy.

Furthermore, this review used Reason’s accident causation model to analyse the data related to the error causation. This model focuses on the system or the environment in which the error occurred, rather than the individual that caused the error, and the random rather than intentional act. However, it is important to note that classifying errors based on this model could be subject to the researchers’ interpretation bias, particularly when the conditions of the error were not thoroughly described. Therefore, mutually exclusive classification of the documented medication errors is not always possible. In addition, well-known causes of medication errors under-reporting such as perceived fears of blame, punishment, or indemnity either by patients, clinicians or administration, consequences of reporting protocol, heavy workload, and lack of time will impede estimating the true prevalence of actual errors reported in the included studies [56]. Therefore, studies that rely on incident reporting databases to identify error rates are likely to be provide underestimation of true prevalence [57].

## Conclusions

This systematic review and meta-analysis suggests that despite their favourable safety profile and relative ease of use compared to VKAs, medication errors with DOACs are common. Future studies should consider data from non-hospital settings and undertake rigorous causality assessment to investigate the link between errors and adverse outcomes. There is a need to promote multidisciplinary working, guideline-adherence, training and education of healthcare professionals, and the use of theory-based and technology facilitated interventions to minimise errors and maximise the benefits of DOAC usage in all settings.

## Supplementary Information

Below is the link to the electronic supplementary material.

Supplementary file1 (PDF 281 KB)

Supplementary file2 (DOC 27 KB)

Supplementary file3 (DOCX 239 KB)

## Data Availability

All data generated or analysed during this study are included in this manuscript.

## Declarations

### Disclaimer

The views expressed are those of the authors and not necessarily those of the NHS, the NIHR, or the Department of Health and Social Care.
