# Supplementary material for: Prevalence, contributory factors and severity of medication errors associated with direct-acting oral anticoagulants in adult patients: a systematic review and meta-analysis
Source: Eur J Clin Pharmacol. 2021 Dec 22;78(4):623–45. doi: 10.1007/s00228-021-03212-y (PMC8926953; doi:10.1007/s00228-021-03212-y)
Supplement: Supplementary file 2 — Supplementary file2 (DOC 27 KB) [file 228_2021_3212_MOESM2_ESM.doc]

**Electronic supplemental material 2**

**Search strategy**

Database: Ovid MEDLINE(R) <1946 to September Week 3 2020>

Search Strategy:

--------------------------------------------------------------------------------

1 exp Medication Errors/ (17416)

2 exp Anticoagulants/ or new oral anticoagulants.mp. (222093)

3 prescribing error*.mp. (615)

4 dispensing error*.mp. (246)

5 drug error*.mp. (327)

6 treatment error*.mp. (371)

7 drug mistake*.mp. (5)

8 therapeutic error*.mp. (278)

9 drug mishap*.mp. (3)

10 medication mistake*.mp. (14)

11 medication mishap*.mp. (12)

12 administration mistake*.mp. (3)

13 dispensing mistake*.mp. (3)

14 prescribing mistake*.mp. (1)

15 wrong drug*.mp. (185)

16 wrong dose*.mp. (176)

17 incorrect drug*.mp. (70)

18 incorrect dose*.mp. (145)

19 medication incident*.mp. (116)

20 preventable adverse drug event*.mp. (152)

21 report prescribing error*.mp. (1)

22 omission error*.mp. (552)

23 transcription error*.mp. (254)

24 discrepancy*.mp. (37482)

25 near miss*.mp. (1966)

26 exp Medication Errors/ (17416)

27 exp Anticoagulants/ (222027)

28 exp Factor X/ or exp Factor Xa Inhibitors/ (12869)

29 oral direct inhibitor*.mp. (33)

30 exp ANTITHROMBINS/ (23147)

31 exp THROMBIN/ (26681)

32 thrombin direct inhibitor*.mp. (2)

33 thrombin inhibitor*.mp. (4385)

34 direct thrombin inhibitor*.mp. (2190)

35 NOAC*.mp. (1768)

36 NOA*.mp. (6509)

37 DOAC*.mp. (1528)

38 TSOAC*.mp. (42)

39 SODA*.mp. (4245)

40 exp DABIGATRAN/ (3146)

41 exp RIVAROXABAN/ (3319)

42 apixaban.mp. (2926)

43 edoxaban.mp. (1217)

44 betrixaban.mp. (145)

45 direct oral anticoagulant*.mp. (2197)

46 target specific oral anticoagulant*.mp. (95)

47 specific oral direct anticoagulant*.mp. (0)

48 novel oral anticoagulant*.mp. (980)

49 new oral anticoagulant*.mp. (1393)

50 Non-vitamin K antagonist oral anticoagulants.mp. (636)

51 NON-WARFARIN ORAL ANTICOAGULANTS.mp. (1)

52 3 or 4 or 5 or 6 or 7 or 8 or 9 or 10 or 11 or 12 or 13 or 14 or 15 or 16 or 17 or 18 or 19 or 20 or 21 or 22 or 23 or 24 or 25 or 26 (58114)

53 27 or 28 or 29 or 30 or 31 or 32 or 33 or 34 or 35 or 36 or 37 or 38 or 39 or 40 or 41 or 42 or 43 or 44 or 45 or 46 or 47 or 48 or 49 or 50 or 51 (253928)

54 52 and 53 (697)

55 limit 54 to yr="2019 -Current" (35)

***************************
