# Supplementary material for: Prevalence, contributory factors and severity of medication errors associated with direct-acting oral anticoagulants in adult patients: a systematic review and meta-analysis
Source: Eur J Clin Pharmacol. 2021 Dec 22;78(4):623–45. doi: 10.1007/s00228-021-03212-y (PMC8926953; doi:10.1007/s00228-021-03212-y)

Subgroup analysis 1: Prescribing error for all indication by setting


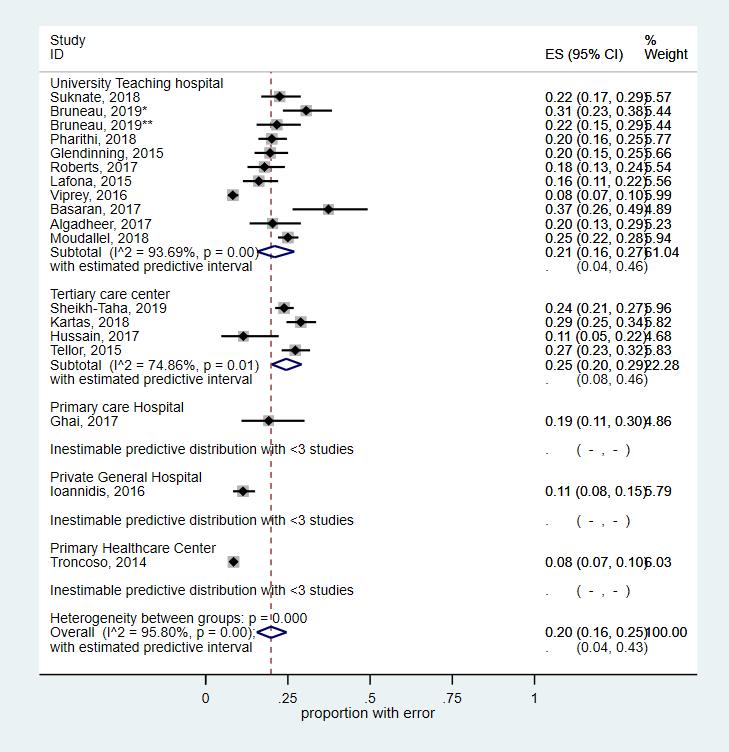


Subgroup analysis 2: Prescribing errors as per indication


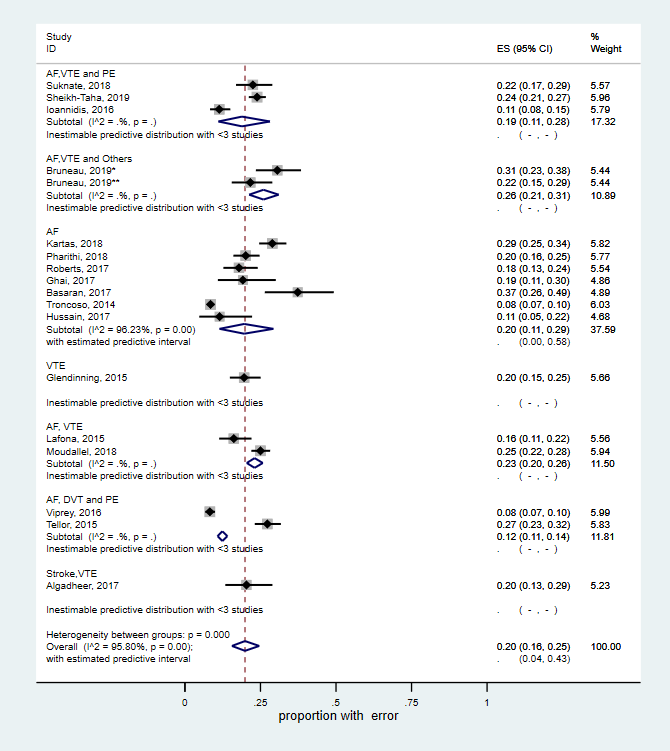


Subgroup analysis 3: Administration error by setting


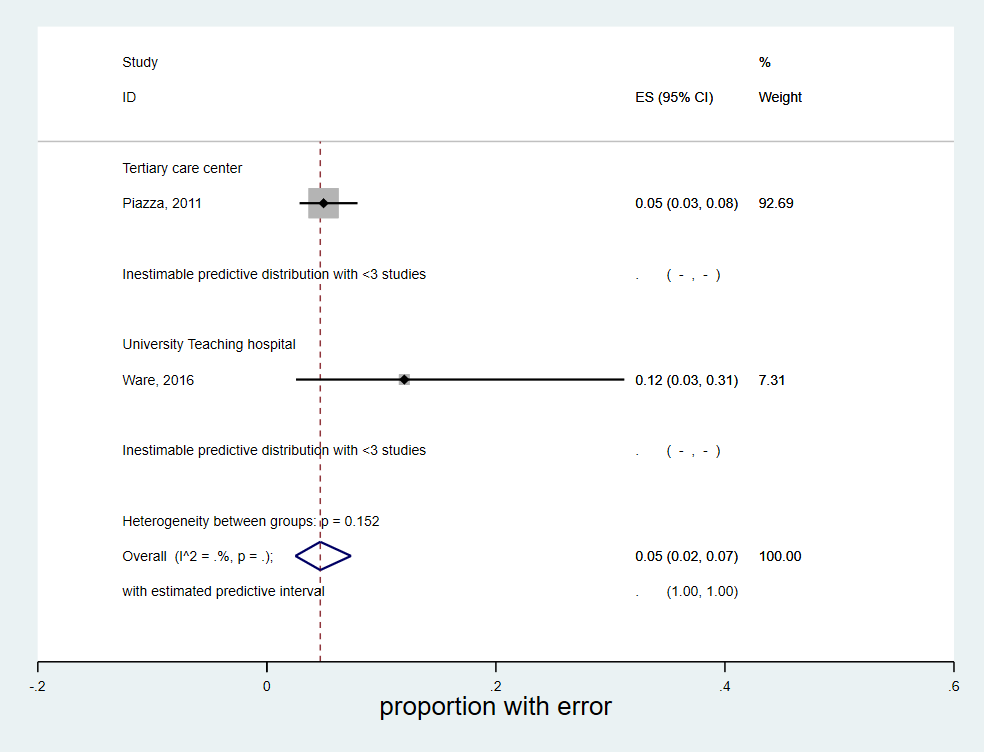


Subgroup analysis 4: Administration error by indication


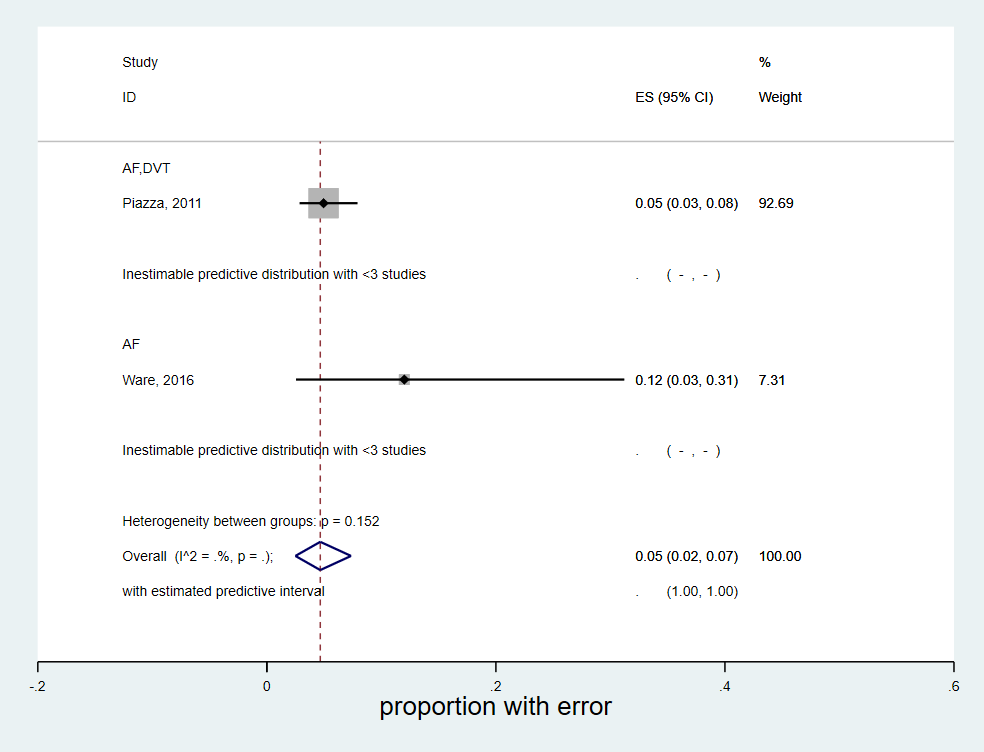

Supplement: Supplementary file 3 — Supplementary file3 (DOCX 239 KB) [file 228_2021_3212_MOESM3_ESM.docx]
